# Ultra-Fast and Sensitive Detection of Non-Typhoidal *Salmonella* Using Microwave-Accelerated Metal-Enhanced Fluorescence (“MAMEF”)

**DOI:** 10.1371/journal.pone.0018700

**Published:** 2011-04-08

**Authors:** Sharon M. Tennant, Yongxia Zhang, James E. Galen, Chris D. Geddes, Myron M. Levine

**Affiliations:** 1 Center for Vaccine Development, University of Maryland School of Medicine, Baltimore, Maryland, United States of America; 2 Institute of Fluorescence, University of Maryland Baltimore County, Baltimore, Maryland, United States of America; Indian Institute of Science, India

## Abstract

Certain serovars of *Salmonella enterica* subsp. *enterica* cause invasive disease (e.g., enteric fever, bacteremia, septicemia, meningitis, etc.) in humans and constitute a global public health problem. A rapid, sensitive diagnostic test is needed to allow prompt initiation of therapy in individual patients and for measuring disease burden at the population level. An innovative and promising new rapid diagnostic technique is microwave-accelerated metal-enhanced fluorescence (MAMEF). We have adapted this assay platform to detect the chromosomal *oriC* locus common to all *Salmonella enterica* subsp. *enterica* serovars. We have shown efficient lysis of biologically relevant concentrations of *Salmonella* spp. suspended in bacteriological media using microwave-induced lysis. Following lysis and DNA release, as little as 1 CFU of *Salmonella* in 1 ml of medium can be detected in <30 seconds. Furthermore the assay is sensitive and specific: it can detect *oriC* from *Salmonella* serovars Typhi, Paratyphi A, Paratyphi B, Paratyphi C, Typhimurium, Enteritidis and Choleraesuis but does not detect *Escherichia coli*, *Pseudomonas aeruginosa*, *Klebsiella pneumoniae*, *Streptococcus pneumoniae*, *Haemophilus influenzae* or *Acinetobacter baumanii*. We have also performed preliminary experiments using a synthetic S*almonella oriC* oligonucleotide suspended in whole human blood and observed rapid detection when the sample was diluted 1∶1 with PBS. These pre-clinical data encourage progress to the next step to detect *Salmonella* in blood (and other ordinarily sterile, clinically relevant body fluids).

## Introduction

*Salmonella*, a genus of more than 2500 serological variants (serovars), includes many organisms that can cause human disease. *Salmonella enterica* subsp. *enterica* Typhi and *S*. Paratyphi A and B, the typhoidal serovars, cause, respectively, typhoid and paratyphoid fevers (enteric fevers), febrile illnesses characterized by infection of the gut-associated lymphoid tissue, liver, spleen, bone marrow and gall bladder and accompanied by a low level bacteremia [1]. Non-typhoidal *Salmonella* (NTS) generally produce a self-limited gastroenteritis (vomiting, fever and diarrhea) in healthy humans [2]–[4]. By contrast, in young infants, the elderly and immunocompromised hosts, NTS can cause severe, fatal disease in both industrialized [4], [5] and developing countries [2], [6]–[12]. Culture-based surveillance for invasive bacterial infections in sub-Saharan Africa have shown that NTS rival *Haemophilus influenzae* type b (Hib) and *Streptococcus pneumoniae* infections in their frequency and severity [6], [9], . Incidence rates of 200–350 cases of invasive NTS infections/10^5^ infections in infants and toddlers have been recorded with case fatality rates of 20–30% [10], [13], [14], [16].

The most common serovars isolated from blood in the USA are *S*. Typhimurium (24%), *S*. Enteritidis (19%) and *S*. Heidelberg (15%) [19]. *S*. Typhimurium and *S*. Enteritidis are also the most commonly NTS serovars isolated from blood and other normally sterile sites from patients in Europe [20], [21] and the United Kingdom [22]. These two serovars, *S*. Typhimurium and *S*. Enteritidis, are particularly prominent in sub-Saharan Africa, where they account for 80–90% of all invasive NTS [6], [9], [10], [12]–[17].

Invasive *Salmonella* spp. are routinely detected by standard blood culture techniques. Culturing blood specimens has become much faster and easier, since the advent of continuously monitoring blood culture instruments such as the BACTEC 9000 systems (Becton Dickinson, Cockeysville, MD, USA) and BacTAlert (BioMerieux, Durham, NC, USA). However, it still takes several days to detect and identify *Salmonella* [23], [24]. Due to the time required for blood culture identification and the fact that many diagnostic laboratories are unable to serotype *Salmonella* spp. themselves, alternative methods of identification of *Salmonella* are being sought [25]. In particular, DNA detection methods such as the polymerase chain reaction (PCR) have been investigated. The food industry routinely uses PCR to detect *Salmonella* in food [26], [27]. There are many reports of PCR primers designed to detect *S*. Typhi from the blood of enteric fever patients [28]–[36]. Furthermore, the sensitivity of PCR has often been found to be higher than that of blood culture [30], [31], [33], [34]. However, PCR has not yet become an established method for diagnosis of typhoid fever [25]. One reason for this may be that although some reports claim high sensitivity, with detection of as few as 10 CFU/ml of blood [35], Wain *et al.*'s prospective study of the concentration of *S*. Typhi in blood of typhoid fever patients showed a median value of 0.3 (range of 0.1 to 399) CFU/ml, well below current PCR-based detection limits [37]. Interestingly, in another study, Wain *et al.* [38] showed that 63% of the *S*. Typhi cells were located in the buffy coat layer (presumably in monocytes and polymorphonuclear leukocytes) and the mean number of bacteria per infected leukocyte was 1.3 CFU/cell. These quantitative studies of Wain *et al.* corroborate a classic early study of Watson [39], who showed a median of 6 CFU/ml of *S*. Typhi in 15 patients with typhoid fever. Gordon *et al.* [40] have shown that NTS in bacteremic patients are present at a similarly low concentration (1 CFU/ml).

A promising new rapid diagnostic technique is microwave-accelerated metal-enhanced fluorescence (MAMEF) [41], [42], which integrates metal-enhanced fluorescence using surface deposited silver nanoparticles (to amplify fluorescence signatures) with low power microwave heating (to accelerate biomolecular recognition events kinetically). MAMEF has detected DNA from *Bacillus anthracis* spores and vegetative cells within 1 minute, which included a ∼30 second spore lysing and sample preparation time [43], [44]. The target was a highly conserved region within the gene encoding protective antigen (PA) [43]. The process was accelerated by using low-power microwave heating. MAMEF has also detected DNA from less than 100 CFU/ml of *Chlamydia trachomatis* in 40 seconds [45]. MAMEF is an ultra-fast, sensitive, and specific assay, using relatively simple but cost-effective technology that can be performed in a 96-well plate [46] and that can be multiplexed [47].

There is a pressing need for a sensitive and specific rapid diagnostic test to detect NTS bacteremia. In this paper, we describe adaption of the ultra-fast, highly sensitive MAMEF technology to detect the *oriC* locus, that we currently use as a *Salmonella*-specific target in PCRs [11], of *Salmonella* spp. which have been rapidly lysed by focused microwave radiation. This work establishes the proof of concept that MAMEF can be used to detect *Salmonella* directly from blood.

## Methods

### Bacterial strains, blood and genomic DNA

We constructed an attenuated *Salmonella* Enteritidis strain CVD 1940 (pGEN206) carrying a deletion in the chromosomal *guaBA* operon of wild-type strain R11 and also carrying plasmid pGEN206 for developing and optimizing microwave lysis and the MAMEF assay (unpublished results). CVD 1940 is unable to synthesize guanine nucleotides and is highly attenuated, therefore making it safer to handle than wild-type *S*. Enteritidis. The parent strain, *S*. Enteritidis R11, was isolated from the blood of a child in Mali [11]. Plasmid pGEN206, which expresses the fluorescent reporter protein GFPuv, encoded by *gfpuv*, was used to monitor efficient lysis of bacteria by fluorescence microscopy. Other *Salmonella* strains tested included *S*. Typhi (2 strains), S. Paratyphi A (2 strains), *S*. Paratyphi B (2 strains), *S*. Paratyphi C (1 strain), *S*. Typhimurium (2 strains), *S*. Enteritidis (2 strains), S. Dublin (2 strains), *S*. Choleraesuis *sensu stricto* (1 strain), *S*. Choleraesuis var. kunzendorf (1 strain) and *S*. Newport (1 strain) that came from *Salmonella* collections at the CVD or the *Salmonella* Reference Laboratory at the Centers for Disease Control and Prevention (Atlanta, GA, USA). Non-*Salmonella* strains used to determine specificity include *Escherichia coli* (2 strains), *Pseudomonas aeruginosa* (1 strain), *Klebsiella pneumoniae* (1 strain) *and Streptococcus pneumoniae* (1 strain each of serovars 6b, 14, 19F and 23) and *Haemophilus influenzae* type b (2 strains). The non-*Salmonella* strains were all from collections stored at the CVD. *Salmonella* spp., *E. coli*, *P. aeruginosa* and *K. pneumoniae* were grown in an animal product-free (APF) LB Lennox medium (APF-LB; Athena Environmental Sciences, Baltimore, MD, USA) at 37°C. Media were supplemented with guanine (0.001% w/v) and 50 µg/ml carbenicillin for growth of CVD 1920 (pGEN206). *S. pneumoniae* was grown on Columbia agar containing 5% Sheep Blood (BD, Franklin Lakes, NJ, USA) or in Brain Heart Infusion (BHI) broth at 37°C with 5% CO_2_. *H. influenzae* was grown on BHI containing 10 µg/ml nicotinamide adenine dinucleotide (NAD) and 10 µg/ml hemin anaerobically using the AnaeroPack System (Mitsubishi Gas Chemical Co., Tokyo, Japan). Whole human blood containing sodium heparin as an anticoagulant was purchased from Innovative Research (Novi, MI, USA). Genomic DNA from *Acinetobacter baumanii* isolates 10 and 11 were obtained from BEI Resources (Manassas, VA, USA).

### DNA methods

Genomic DNA was isolated using the WIZARD SV Genomic DNA Purification system (Promega, Madison, WI, USA) according to the manufacturer's instructions. DNA was visualized by agarose gel electrophoresis and staining with ethidium bromide. DNA released from lysed bacteria was visualized by agarose gel electrophoresis following concentration by ethanol precipitation. Briefly, 450 µl of lysate were concentrated by ethanol precipitation. The DNA pellet was resuspended in 20 µl TE (10 mM Tris and 1 mM EDTA [pH 8.0]) and the entire volume was electrophoresed on a 0.9% agarose gel.

### Deposition of gold triangles on glass substrates to lyse *Salmonella*

**Figure 1:**
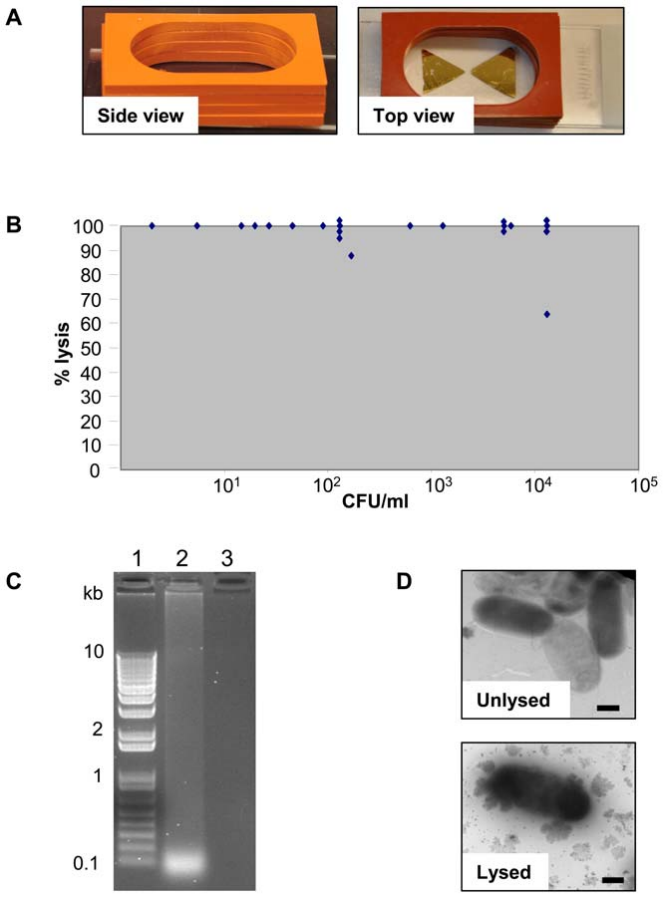
Lysis of *S*. Enteritidis. (A) Gold lysing triangles with bowtie configuration. (B) Lysis of biologically relevant concentrations of CVD 1920 (pGEN206). 2 CFU/ml to 1.3×10^4^ CFU/ml were lysed using gold lysing triangles and heated for 13 s on high power in a GE microwave Model No. JE2160BF01. (C) Agarose gel of DNA released from lysed CVD 1920 (pGEN206). An overnight culture of bacteria (1.9×10^10^ CFU/ml) was lysed using gold lysing triangles and microwave radiation. Lane 1, 1 kb Plus DNA ladder (Invitrogen); 2, lysed bacteria; 3, unlysed bacteria. (D) Transmission electron micrographs of lysed and unlysed CVD 1920 (pGEN206). Bar = 500 nm.

Glass microwave slides were covered with a mask (12.5 mm in size with a 1 mm gap between two triangles), leaving a bowtie region exposed. Equilateral gold triangles of 12.5 mm were subsequently deposited onto glass microscope slides through the mask using a BOC Edwards 306 vacuum deposition with vacuum 3.0×10^−6^ Torr, with a deposition rate of ∼1 A/s. Four layers of self-adhesive silicon isolators (D 2.5 mm) were placed over the gold bowtie region to create a sample well (see Figure 1A).

### Measurement of temperature over the gold bowtie

Since sapphire transmits infrared radiation, it is an ideal substrate for thermal imaging experiments. A slide coated with gold triangles and containing water in the sample well was covered by a sapphire plate. Using this sapphire and metal sandwich configuration, we determined the average temperature increase of the water in proximity to the metal. The optical configuration consisted of a microwave cavity with a 1 inch diameter opening at the base, a gold mirror, and a thermal imaging camera (Silver 420 M; Electrophysics Corp, Fairfield, NJ, USA) that was equipped with a lens that provided a resolution of approximately 300 µm (Figure S1). The clear sapphire plate of the sandwich geometry was fixed to the base of the microwave cavity opening. A gold mirror was positioned such that the image of the opening was reflected onto the thermal camera. Thermal imaging data was recorded at 100 frames/second before, during and after the application of microwave pulses.

### Lysis by microwave radiation

Bacteria were lysed using gold bowtie deposits on a glass slide and heating in a GE microwave Model No. JE2160BF01, kW:1.65 (M/W), for 13 seconds on maximum power. 2 ml bacterial suspensions were placed into wells sterilized by rinsing with 70% ethanol and air-drying. Overnight cultures of bacteria were diluted to the required concentration in APF-LB and lysed. Viable counts of unlysed samples were performed by spread-plating up to 200 µl of bacterial suspension on agar plates or by using the pour plate method (5 ml of the bacterial suspension was added to 20 ml of molten agar and poured into a petri dish). Viable counts of lysed samples were performed by spread-plating 100–200 µl on APF-LB and incubating the remaining suspension at 37°C overnight. A sample was considered to be fully lysed if the broth showed no turbidity.

### Transmission Electron Microscopy (TEM)

TEM images of lysed bacteria were taken using an Electron Microscope Tecnai T12 microscope. Samples were drop cast onto Formvar carbon films on copper grids (400 mesh) by placing a droplet of a 10-µl aqueous sample solution on a grid. The grid was air-dried for 24 h.

### Formation of silver island films (SiFs) on glass substrates

SiFs, which provide the metal component of ‘metal-enhanced fluorescence’, were prepared as previously published [48]. In a typical SiFs preparation, a solution of silver nitrate (0.5 g in 60 ml of deionized water) was put in a clean 100 ml glass beaker. 200 µl of freshly prepared 5% (w/v) sodium hydroxide solution and 2 ml of ammonium hydroxide were added to a continuously stirred silver nitrate solution at room temperature. Subsequently, the solution was cooled to 5°C by placing the beaker in an ice bath, followed by soaking the Silane-prep™ glass slides in the solution and adding a fresh solution of *D*-glucose (0.72 g in 15 ml of water). The temperature of the mixture was then allowed to warm to 40°C. As the color of the mixture turned from yellow-green to yellowish brown, the slides were removed from the mixture, washed with water, and sonicated for 1 min at room temperature.

### Anchor and fluorescent probes

We have successfully used previously published primers to detect *oriC* of *Salmonella* in several PCRs [11], [49]. We therefore decided to use this region as a target for our *Salmonella* MAMEF-based assay. Probes specific for the *oriC* locus of *Salmonella* spp. were designed using the *oriC* sequence from *S*. Typhimurium LT2 (GenBank accession no. AE006468). Anchor probe (5′ GTTTTTCAACCTGTTTTGCGCC 3′), fluorescent probe (5′ CTTTCAGTTCCGCTTCTAT 3′) and a synthetic target oligonucleotide (5′ATAGAAGCGGAACTGAAAGGCGCTGGCGCAAAACAGGTTG 3′) were purchased from Sigma-Aldrich (St. Louis, MO, USA). 16 nucleotides of the anchor probe bind to the *oriC* target. The remaining nucleotides consist of a guanine to which a thiol group is added and 5 T's for flexibility of the probe following binding to the glass slide. The fluorescent probe possesses a TAMRA dye attached to the first nucleotide at the 5′ end.

### Preparation of MAMEF assay platform for detection of *Salmonella* DNA

SiFs-deposited glass slides were coated with self-adhesive silicon isolators, containing oval wells (2.0 mm D×32 mm L×19 mm W) prior to the assay fabrication and subsequent fluorescence experiments. 1 µM of thiolated anchor probe was incubated overnight at 4°C on the surface of SiFs-deposited glass slides in 1× TE Buffer. The thiolated anchor probe was covalently linked to SiFs via well-established self-assembled monolayer chemistry [50].

### MAMEF-based *Salmonella* DNA assays

The MAMEF-based DNA capture assay was performed using 1 ml lysed *Salmonella* mixed with 1 ml TAMRA-labeled fluorescent probe overlaid onto the SiFs containing bound anchor probe and incubated for 30 seconds in a microwave cavity (a 0.7 cu ft, GE Compact Microwave Model: JES735BF, max power 700 W). The power setting of the microwave cavity was set to 2 (which corresponded to 140 W over the entire cavity).

### Fluorescence Spectroscopy

Fluorescence emitted by the MAMEF-based DNA capture assay was measured using a 532 nm diode laser and a Fiber Optic Spectrometer (HD2000) from Ocean Optics, Inc. by collecting the emission intensity through a notch filter (532 nm).

## Results

### Lysis of *Salmonella*

We tested a variety of configurations of gold deposited on glass slides and found that the gold bowtie configuration was the best in terms of its ability to effectively lyse *Salmonella* when heated in a microwave (Figure 1A). Figure S1 shows that the temperature of liquid at the point where the two triangles apex is raised during microwave heating. Overnight cultures of *S*. Enteritidis CVD 1920 (pGEN206) were diluted to biologically relevant concentrations (up to 10^4^ CFU/ml) in APF-LB and 2 ml were lysed by microwave radiation. As can be seen in Figure 1B, a range of 2 CFU/ml to 1.3×10^4^ CFU/ml could be efficiently lysed (98%±8% lysis; mean ± standard deviation) using gold lysing triangles and heating in a microwave. DNA released from an overnight culture (1.9×10^10^ CFU/ml) of CVD 1920 (pGEN206) lysed using gold lysing triangles and a microwave was fragmented into a range of sizes with most of the fragments around 100 bp, as shown in Figure 1C. Examination of lysed bacteria by electron microscopy showed bacteria with blurred edges surrounded by clumps of lysed debris; bacteria from unlysed samples showed distinct edges against a clear background (Figure 1D).

### Detection of a synthetic *oriC* oligonucleotide by MAMEF

**Figure 2:**
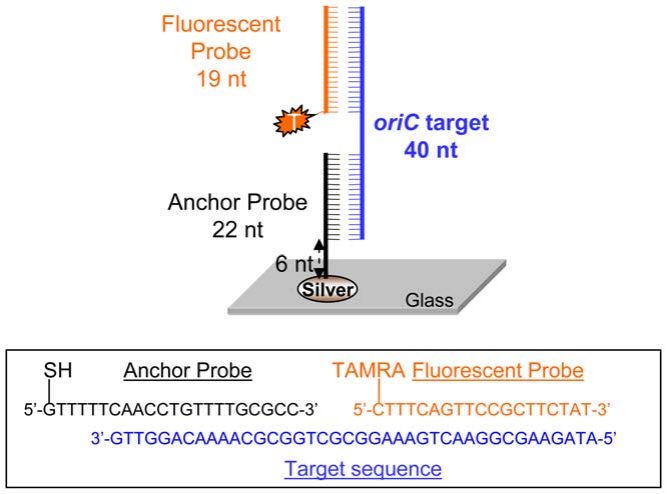
Schematic representation of anchor and fluorescent probes binding to *Salmonella oriC* target DNA.

**Figure 3:**
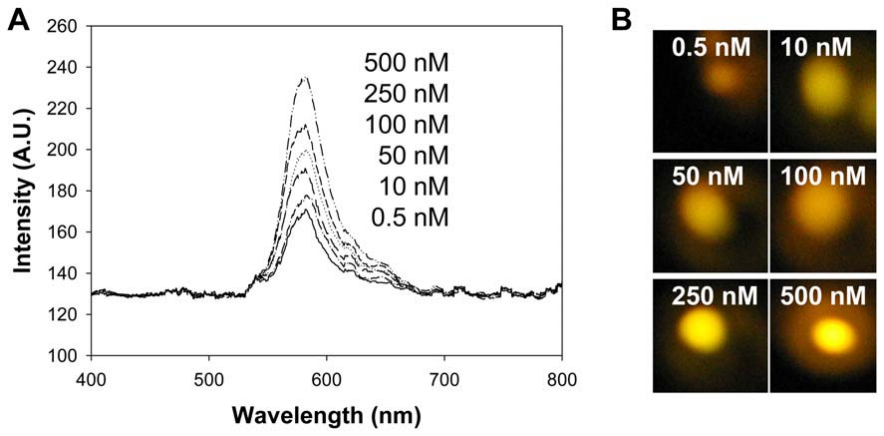
Detection of various concentrations of a synthetic *oriC* oligonucleotide by MAMEF. (A) The graph shows the increase in intensity as the concentration of oligonucleotide increases. (B) The actual fluorescent signal produced at each concentration. Excitation = 532 nm, Emission = 575 nm.

As mentioned earlier, *oriC* is used as a target in several *Salmonella* PCRs [11], [51], [52]. Figure S2 shows the primer binding sites in *S*. Typhimurium LT2 (GenBank Accession No. AE006468) of two different *oriC* primer pairs, as well as the location in bold of the segment of DNA targeted by our fluorescent and anchor probes. This *oriC* target DNA is conserved in all serovars of *Salmonella enterica* subsp. *enterica* except for *S*. Agona, *S*. Gallinarum and *S*. Dublin (Figure S2). We therefore designed anchor and fluorescent probes so that they can detect all *Salmonella enterica* subsp. *enterica* serovars including *S*. Agona, *S*. Gallinarum and *S*. Dublin. Figure 2 shows a schematic diagram of the anchor and fluorescent probes binding to the target DNA. We first tested the ability of the probes to detect a synthetic oligonucleotide target. As can be seen in Figure 3, the target DNA was detected in a concentration-dependent manner, successfully detecting *oriC* at concentrations ranging from 500 nM all the way down to 0.5 nM.

### Detection of DNA released from lysed *Salmonella*

**Figure 4:**
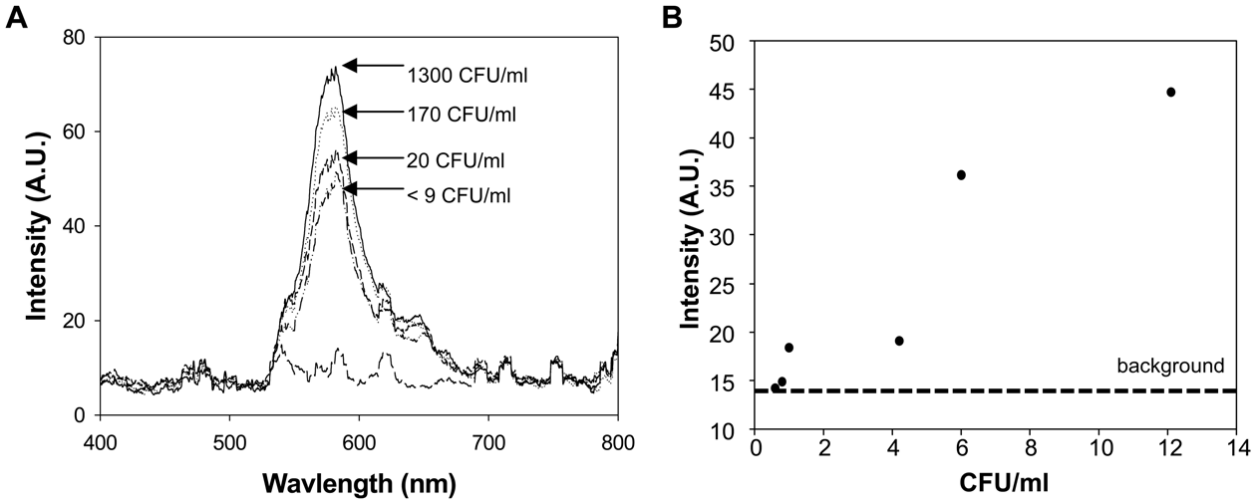
Detection of DNA released from microwave-lysed CVD 1920 (pGEN206) suspended in bacteriological media. Serial dilutions from two independent samples were examined: A) 10-fold serial dilutions of a 10^3^ CFU/ml suspension and B) 2-fold serial dilutions of a 12 CFU/ml suspension.

Once lysis and detection were optimized, we performed the two methods sequentially to detect DNA using MAMEF from *Salmonella* that had been lysed by microwave radiation. Overnight cultures of CVD 1920 (pGEN206) were diluted to biologically relevant concentrations (up to 10^3^ CFU/ml) in APF-LB and 2 ml were lysed by microwave radiation. 1 ml of the lysed bacteria was tested by MAMEF. We tested serial dilutions of two independent samples, with each dilution lysed separately. The first consisted of 10-fold serial dilutions of a 10^3^ CFU/ml suspension and lysis results are shown in Figure 4A. The second dilution series consisted of 2-fold serial dilutions of a 12 CFU/ml suspension, with results shown in Figure 4B. Once again, the intensity of the fluorescence signal was concentration dependent and we were able to detect *S*. Enteritidis at concentrations down to 1 CFU/ml (Figure 4B).

### Specificity of the *Salmonella* MAMEF assay

To demonstrate that the assay is specific for detecting only *Salmonella* DNA, we tested DNA from a variety of *Salmonella* serovars and other bacteria commonly isolated from blood. Sixteen *Salmonella* strains of various serovars were suspended in APF-LB media at 10^3^–10^4^ CFU/ml and 2 ml of each suspension were lysed using microwave radiation; every strain was lysed (93%±16%, mean ± standard deviation). 1 ml of the lysed sample was then assayed by MAMEF and a fluorescent signal was observed for every strain tested (Table 1). When 2 ml of 10^4^ CFU/ml suspensions of *E. coli*, *P. aeruginosa* and *K. pneumoniae* were lysed by microwave lysis and tested by MAMEF, no fluorescence signal was observed. We also tested genomic DNA (diluted to 100 pg/ml) from these 3 species as well as DNA from *S. pneumoniae*, *H. influenzae* and *A. baumanii* and did not observe any detection (Table 1).

**Table 1:** Detection of *Salmonella* and non-*Salmonella* strains that are commonly found in blood.

Species/serovar	Number of strains tested	Method of DNA extraction	Detection
*Salmonella enterica subsp. enterica* serovars			
Typhi	2	Microwave lysis	+
Paratyphi A	2	Microwave lysis	+
Paratyphi B	2	Microwave lysis	+
Paratyphi C	1	Microwave lysis	+
Typhimurium	2	Microwave lysis	+
Enteritidis	2	Microwave lysis	+
Dublin	2	Microwave lysis	+
Choleraesuis (sensu stricto)	1	Microwave lysis	+
Choleraesuis var. Kunzendorf	1	Microwave lysis	+
Newport	1	Microwave lysis	+
Non-*Salmonella*			
*E. coli*	2	Microwave lysis and genomic DNA isolation	−*
*P. aeruginosa*	1	Microwave lysis and genomic DNA isolation	−*
*K. pneumoniae*	1	Microwave lysis and genomic DNA isolation	−*
*S. pneumoniae*	4	Genomic DNA isolation	−*
*H. influenzae*	2	Genomic DNA isolation	−*
*A. baumannii*	2	Genomic DNA isolation	−*

*Genomic DNA was assayed in 100 µl wells.

### Detection of *Salmonella* DNA in blood

**Figure 5:**
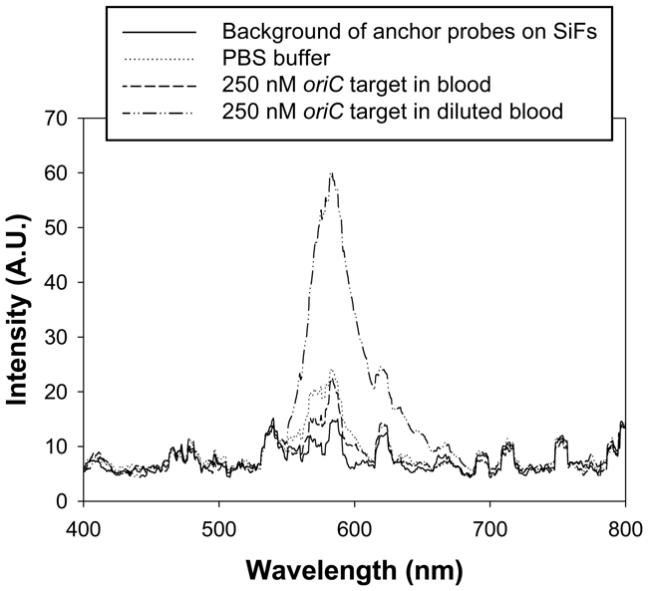
MAMEF-based detection of a synthetic *oriC* oligonucleotide suspended in whole human blood. Whole human blood was spiked with the *oriC* oligonucleotide (250 nM) and tested by MAMEF or diluted 1∶1 with PBS and then tested by MAMEF.

The overall goal of our research efforts is to detect *Salmonella* directly from blood. We have performed some preliminary experiments using blood spiked with the synthetic *oriC* oligonucleotide and were not able to observe fluorescence (Figure 5). However, when the spiked blood sample was diluted with an equal volume of PBS, the synthetic *oriC* target was readily detected by MAMEF.

## Discussion

We have developed a MAMEF-based *Salmonella* assay capable of lysing and detecting 1 CFU suspended in 1 ml of bacteriological medium. The time to detection (excluding processing time) was only 30 seconds. This level of speed and detection limit greatly surpasses all currently available assays. For example, Nga *et al.* [53] have recently described a multiplex real-time PCR assay that targets *S*. Typhi and *S*. Paratyphi A. The sensitivity of the assay on blood samples was low with only 42% sensitivity for *S*. Typhi and 39% sensitivity for *S*. Paratyphi A. This poor sensitivity is most likely due to the poor detection limit of the assay. The authors prepared serial dilutions of *S*. Typhi in blood and observed detection limits of 250 CFU/ml for their real-time assay and 25 CFU/ml for detection by blood culture. The authors concede that identification of invasive *Salmonella* by PCR is not a practical approach.

Sensitivity of nucleic acid-based detection can be markedly increased by introducing an incubation step but only at the cost of greatly increasing the duration of the assay and its adaptation to becoming a point of care diagnostic test. For example, Zhou and Pollard [54] overcame low sensitivity due to small sample volumes by including a 3-hour incubation step in tryptone soya broth containing 2.4% ox bile, prior to detection of *S*. Typhi by PCR. They were able to achieve a detection limit of 0.75 CFU per ml of blood. However, the entire protocol takes almost 8 hours to complete. Moreover, the need for an incubation step in culture media means that this is no longer amenable to being a point of care diagnostic test.

Similarly, by incorporating an overnight enrichment step, a fluorescence *in situ* hybridization (FISH) method for the detection of *Salmonella* spp. using a novel peptide nucleic acid (PNA) probe has achieved 100% sensitivity and 100% specificity and can detect 1 CFU per 10 ml of blood [55]. Because of the lengthy (overnight) enrichment step, this assay is also neither rapid nor adaptable to become the much needed point of care diagnostic for invasive *Salmonella* disease.

One molecular assay that does not require an enrichment step is the Lightcycler Septi*Fast* Test MGRADE kit (Roche Diagnostics, Germany), which is a commercial real-time PCR assay. This kit detects and identifies the 25 most common pathogens known to cause bloodstream infections directly from whole blood in <6 hours but does not target *Salmonella* [56]. In serial experiments performed on EDTA-blood samples spiked with different concentrations of bacterial and fungal reference organisms, hit rates of 70–100% were achieved for 23 out of 25 organisms at 30 CFU/ml, but for only 15 out of 25 organisms at 3 CFU/ml. These results suggest that the assay may not be as sensitive as blood culture which has a theoretical sensitivity of 1 CFU. However, two studies indicate that the Lightcycler Septi*Fast* is more sensitive than blood culture, as it was able to detect target DNA in several samples which were negative by blood culture [57], [58]. Disadvantages of this method are that it includes a sample preparation step that requires the use of a centrifuge and the time taken to detection.

Recently, a MAMEF method showing detection of biotinylated BSA (b-BSA) was used to establish that MAMEF can be used to detect targets in complex biological samples such as blood [59]. In this study, phosphate buffer containing b-BSA was mixed with whole blood in a 1∶1 ratio, and the target protein was detected using fluorophore-labeled avidin in 1 min. These findings and our own results show that detection of protein and DNA targets in blood is possible though presently the blood needs to be diluted to enable detection. We are currently investigating various methods to lyse red and white blood cells to reduce some of the viscosity of the liquid which we believe is impeding mass transport of the biological components to the surface during microwave-acceleration, and therefore overall fluorescence detection, and to release bacteria within the white blood cells.

The key points of the MAMEF technology that make it attractive for detection of pathogens in blood include: 1) it is a rapid and highly sensitive method; 2) the technology has been multiplexed, so that in one sample well, three DNA or protein targets can presently be identified within 20–40 seconds; 3) detection of well fluorescence can be accomplished by a variety of standard inexpensive sample well-reader technologies; 4) the assay platform requires no washing steps whatsoever to remove excess fluorescent probe or labeled DNA/antibody; 5) chambers that are disposable to minimize cross-contamination; 6) absence of centrifugation steps; and 7) the assay can be made quantitative by comparing levels of fluorescence to a standard curve. These attributes make it possible for the assay to be developed ultimately into a point-of-care device that can be used by people with minimal training.

The novel findings of this study are: 1) we have developed a sensitive and specific MAMEF-based *Salmonella* assay; 2) we have shown detection in 1 ml sample volumes which is greater than previously reported (and which provides the proof-in-principle that large volumes can be tested by MAMEF); 3) we can lyse and detect *Salmonella* without any centrifugation or washing steps; and 4) detection in blood is possible. We anticipate that we will be able to develop a multiplex MAMEF-based *Salmonella* assay that can efficiently detect the chromosomal *oriC* from blood-borne *Salmonella* and further determine whether the serovar is *S*. Typhimurium or *S*. Enteritidis, the two most commonly isolated NTS from invasive sites.

## Supporting Information

Figure S1 **Thermal imaging of gold bowties.** A) Optical scheme for thermal imaging of gold bowties and sapphire sample geometries. B) Temperature of water at the apex of the gold bowtie triangles over time. The graph shows the mean intensity temperature over a 100×100 pixel region.
(TIF)

Figure S2 **Location of** ***oriC*** **primer binding sites and MAMEF target DNA in the** ***S*** **. Typhimurium genome.** Bold, segment of *S*. Typhimurium LT2 DNA (5′ to 3′) targeted by *Salmonella* MAMEF assay; underlined, primer binding sites of *oriC* primers described by Woods *et al.* [52] ; and italicized, primer binding sites of *oriC* primers described by Widjojoatmodjo *et al.* [51].
(TIF)
