# Supplementary figures and images for: Ultra-Fast and Sensitive Detection of Non-Typhoidal Salmonella Using Microwave-Accelerated Metal-Enhanced Fluorescence (“MAMEF”)
Source: PLoS One. 2011 Apr 8;6(4):e18700. doi: 10.1371/journal.pone.0018700 (PMC3073000; doi:10.1371/journal.pone.0018700)

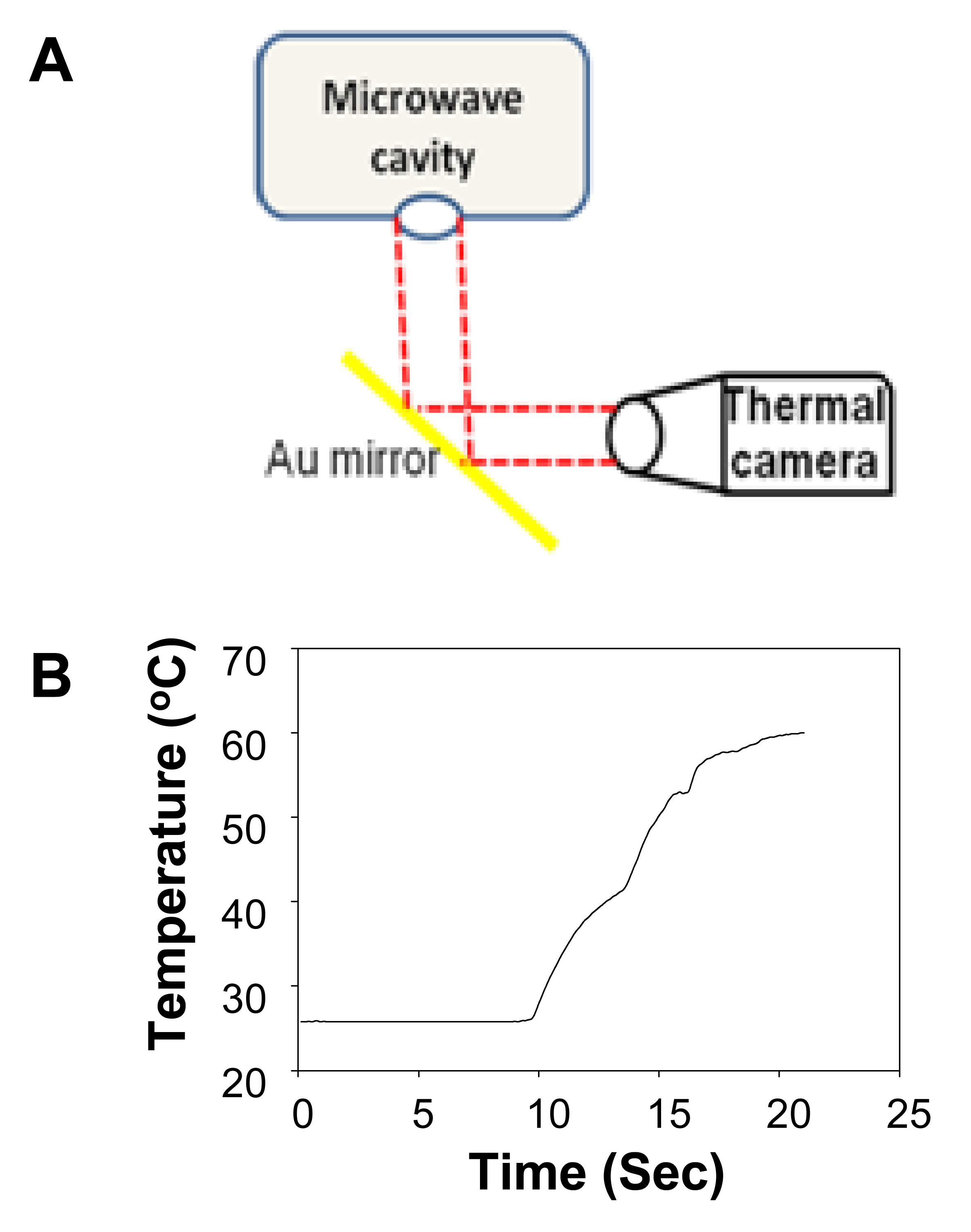

Supplement: Figure S1 — Thermal imaging of gold bowties. A) Optical scheme for thermal imaging of gold bowties and sapphire sample geometries. B) Temperature of water at the apex of the gold bowtie triangles over time. The graph shows the mean intensity temperature over a 100×100 pixel region. (TIF) [file pone.0018700.s001.tif]

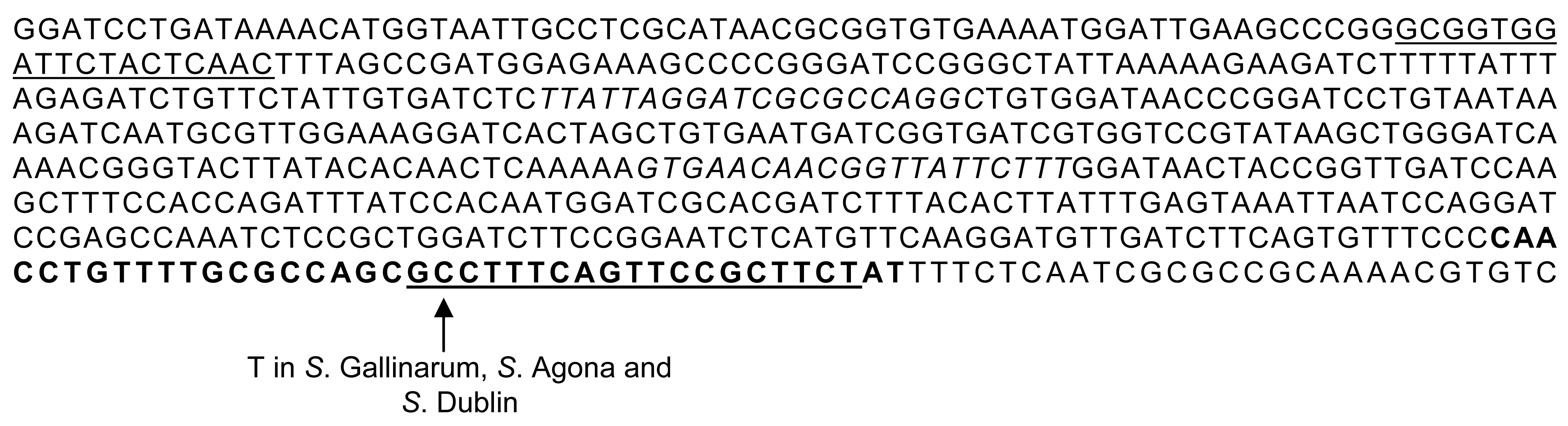

Supplement: Figure S2 — Location of oriC primer binding sites and MAMEF target DNA in the S . Typhimurium genome. Bold, segment of S. Typhimurium LT2 DNA (5′ to 3′) targeted by Salmonella MAMEF assay; underlined, primer binding sites of oriC primers described by Woods et al. [52] ; and italicized, primer binding sites of oriC primers described by Widjojoatmodjo et al. [51]. (TIF) [file pone.0018700.s002.tif]
